# Homolytic substitution at phosphorus for C–P bond formation in organic synthesis

**DOI:** 10.3762/bjoc.9.143

**Published:** 2013-06-28

**Authors:** Hideki Yorimitsu

**Affiliations:** 1 Department of Chemistry, Graduate School of Science, Kyoto University, Sakyo-ku, Kyoto 606-8502, Japan; 2 ACT-C, Japan Science and Technology Agency, Sakyo-ku, Kyoto 606-8502, Japan

**Keywords:** addition, free radical, homolysis, phosphine, radical, substitution

## Abstract

Organophosphorus compounds are important in organic chemistry. This review article covers emerging, powerful synthetic approaches to organophosphorus compounds by homolytic substitution at phosphorus with a carbon-centered radical. Phosphination reagents include diphosphines, chalcogenophosphines and stannylphosphines, which bear a weak P–heteroatom bond for homolysis. This article deals with two transformations, radical phosphination by addition across unsaturated C–C bonds and substitution of organic halides.

## Introduction

**Scheme 1:**
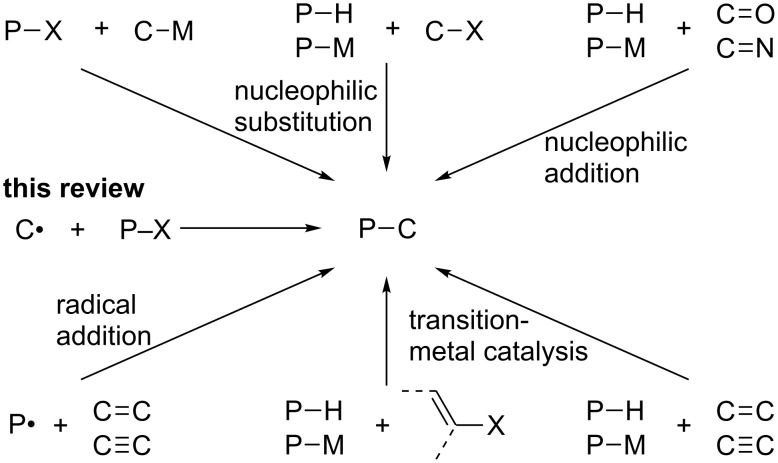
Representative C–P bond-forming reactions.

Organophosphorus compounds constitute an important class of compounds in a wide range of applications in organic chemistry, as reagents, intermediates, ligands, bioactive agents, and functional materials [1–4]. The synthesis of organophosphorus compounds has therefore been extensively investigated (Scheme 1). Classical methods to form a C–P bond include ionic reactions such as nucleophilic substitution of P–X compounds with organometallic reagents, nucleophilic substitution of alkyl halides with phosphorus nucleophiles, and nucleophilic addition to polar unsaturated bonds. Recent advances in transition-metal catalysis have realized catalytic cross-coupling reactions of aryl halides with H–P compounds [5–7] and catalytic addition to nonpolar unsaturated carbon–carbon bonds [8–11]. In the field of radical chemistry, the addition of phosphorus radicals, mainly from H–P compounds, onto carbon–carbon multiple bonds [12–15] has held a special position since they provide transformations unattainable by polar reactions.

**Scheme 2:**
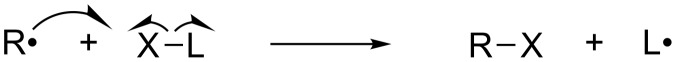
General equation of homolytic substitution.

Homolytic substitution is a reaction in which a radical (R•) attacks a saturated atom (X) in a molecule with the liberation of a leaving radical (L•) from the atom (Scheme 2). Homolytic substitution at halogen and chalcogen atoms is well known to proceed and hence has been widely used in organic synthesis [16–19]. In contrast, applications of homolytic substitution to C–P bond formation have been rarely explored. With the growing importance of organophosphorus compounds, increasing attention has been paid to homolytic substitution at phosphorus. The new tool for C–P bond formation has achieved interesting transformations that ionic reactions cannot. This review summarizes homolytic substitution at phosphorus for C–P bond formation in organic synthesis while the relevant mechanistic studies are found in the literature [19–21]. This review deals with two transformations, radical phosphination by addition across unsaturated C–C bonds and substitution of organic halides.

## Review

### Radical addition of phosphination agents

**Scheme 3:**
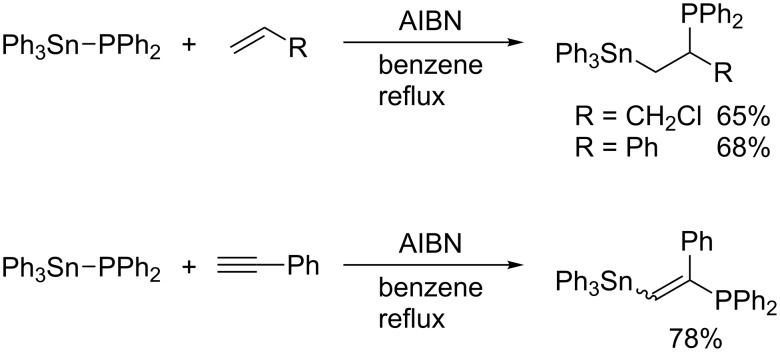
Addition of diphenyl(triphenylstannyl)phosphine.

**Scheme 4:**
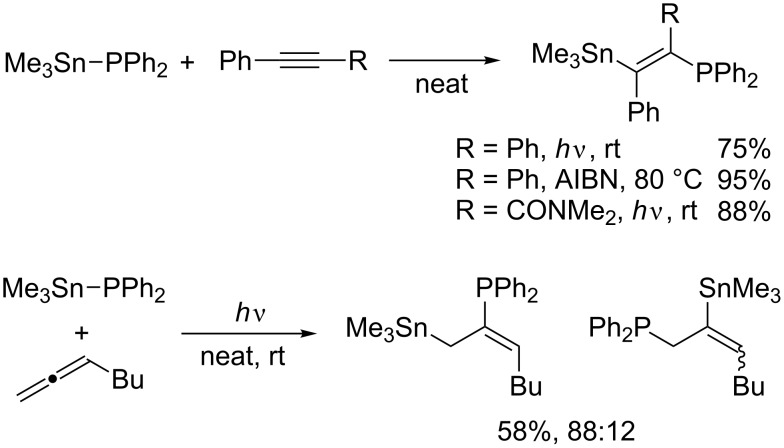
Addition of diphenyl(trimethylstannyl)phosphine.

**Scheme 5:**
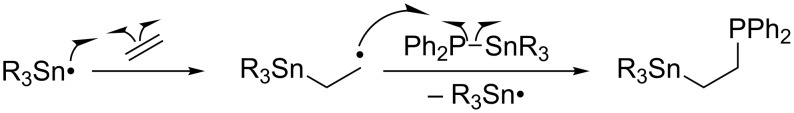
Plausible mechanism of addition of R_3_Sn–PPh_2_.

Stannylphosphines of the type R_3_Sn–PR’_2_ are known to undergo radical addition to carbon–carbon unsaturated bonds. Schumann reported the addition of diphenyl(triphenylstannyl)phosphine to allyl chloride, styrene, and phenylacetylene (Scheme 3) [22–23]. The addition is most likely to proceed via a radical process as the absence of AIBN leads to considerable decreases in yield. Mitchell then reported that diphenyl(trimethylstannyl)phosphine reacts not only with terminal alkynes but also with internal alkynes and allenes (Scheme 4) [24–25]. It is noteworthy that the regioselectivity of the radical addition to propynamide is opposite to that of the relevant ionic Michael addition. Considering the regioselectivity, these addition reactions naturally involve C–P bond formation by homolytic substitution at phosphorus (Scheme 5). Studer recently reported similar silylphosphination of phenyl vinyl sulfone with Me_3_Si–PPh_2_ [26].

**Scheme 6:**
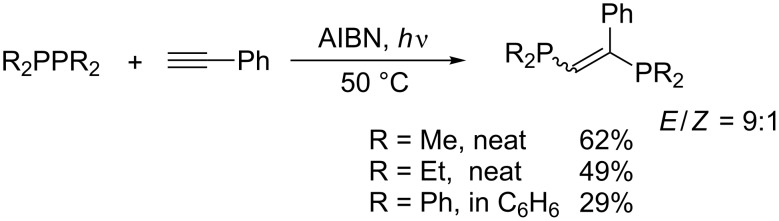
Addition of tetraorganodiphosphines to phenylacetylene.

**Scheme 7:**
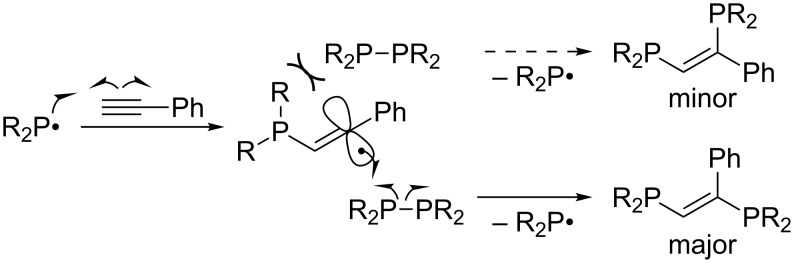
Plausible mechanism of *anti*-diphosphination.

Tzschach reported that tetraorganodiphosphines R_2_P–PR_2_ add to phenylacetylene under UV irradiation or upon heating in the presence of AIBN (Scheme 6) [27]. The reaction consists of the addition of a diorganophosphanyl radical to phenylacetylene and the homolytic substitution of tetraorganodiphosphine with the resulting vinyl radical to afford the adduct and to regenerate the initial diorganophosphanyl radical (Scheme 7). The high *E* selectivity is attributable to kinetic control of the homolytic substitution, where R_2_P–PR_2_ preferentially approaches the vinyl radical from the roomier side. Although the transformation looks useful to construct an (*E*)-1,2-diphosphanylethene skeleton, the scope of alkyne is limited to phenylacetylene and the reactions result in unsatisfactory yields because of the instability of the products as well as the diphosphines in air.

**Scheme 8:**
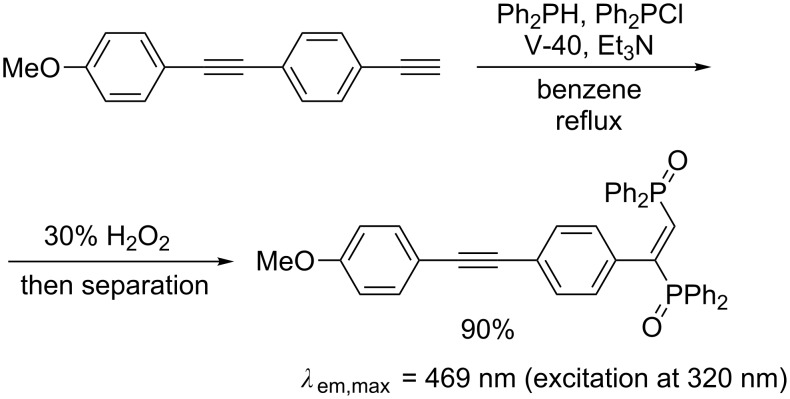
Radical diphosphination for synthesizing fluorescent material.

A more general, facile, and reliable method for diphosphination was later reported by Yorimitsu and Oshima, which utilizes diphosphines generated in situ from chlorophosphine and hydrophosphine in the presence of triethylamine [28]. A variety of terminal alkynes undergo the radical diphosphination (Table 1). The diphosphination was applicable to the synthesis of a new push–pull-type molecule that emits blue fluorescence (Scheme 8). The initially formed diphosphanylethylene derivatives are not very stable in air, and therefore sulfidation or oxidation was performed to accurately assess the efficiency of the diphosphination reactions.

**Table 1:** Radical *anti*-selective diphosphination of terminal alkynes.

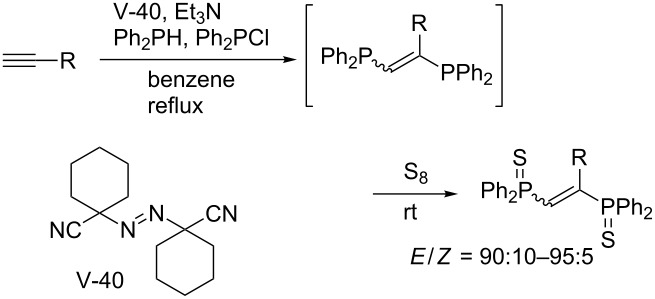	

R	Yield (%)

C_10_H_21_	84
Ph	87
C_6_H_4_-*p*-OMe	89
C_6_H_4_-*p*-CO_2_Me	95
C_6_H_4_-*p*-I	83
C_6_H_4_-*p*-COMe	96
(CH_2_)_3_OBn	78
(CH_2_)_6_CO_2_Et	86
(CH_2_)_9_SCOMe	80
(CH_2_)_9_Cl	86

Ogawa independently reported similar diphosphination under UV irradiation (Table 2) [29–30]. The reactions favor the formation of *Z* isomers, which results from photoinduced isomerization of initially formed *E* isomers. Ogawa’s diphosphination is thus potentially useful for the synthesis of (*Z*)-1,2-diphosphanyl-1-alkenes, which can serve as bidentate ligands.

**Table 2:** Photoinduced radical diphosphination of terminal alkynes.

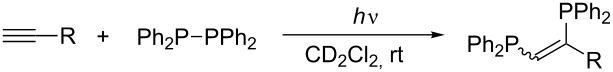			

R	Time (h)	Yield (%)	*E*/*Z*

CH_2_CH_2_CHMe_2_	39	62	18:82
(CH_2_)_3_Cl	18	55	42:58
Ph	1	45	<1:99

Morse developed photoinduced addition of tetrafluorodiphosphine to alkenes and alkynes in the gas phase (Table 3) [31–34]. The addition provides a series of intriguing bidentate phosphine ligands. The addition to alkynes yields 1:1 mixtures of *E*/*Z* isomers. Due to the high reactivity of a difluorophosphanyl radical, considerable polymerization takes place unless substrates or olefinic products are reasonably inert.

**Table 3:** Photoinduced radical diphosphination with tetrafluorodiphosphine.

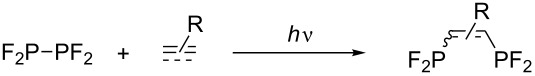	

Product	Yield (%)

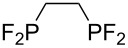	50
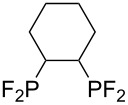	62
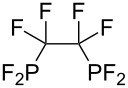	<10
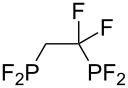	13
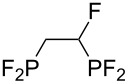	52
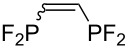	0
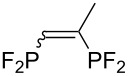	5
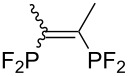	10
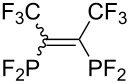	65
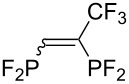	25

**Scheme 9:**
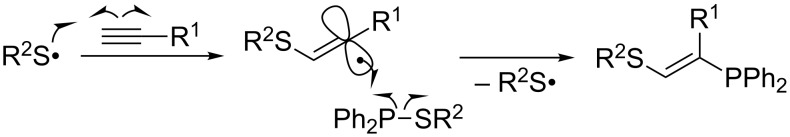
Mechanism of thiophosphination with diphenyl(organosulfanyl)phosphine.

**Scheme 10:**
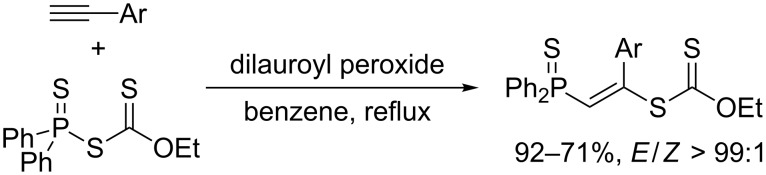
Thiophosphination with *S*-thiophosphinyl *O*-ethyl dithiocarbonate.

Yorimitsu and Oshima reported radical addition of a P–S bond across alkyne by using diphenyl(organosulfanyl)phosphine (Table 4) [35]. The addition proceeds mainly in an *anti* fashion to afford the adducts bearing a sulfanyl group at the terminal carbon and a phosphanyl group at the internal carbon. The reaction mechanism is similar to that in Scheme 7 (Scheme 9). The regioselective outcome suggests that the homolytic substitution occurs exclusively at phosphorus, not at sulfur. A sulfanyl radical is liberated to add the terminal carbon of alkyne. To reverse the regioselectivity in radical addition of a P–S bond, *S*-thiophosphinyl *O*-ethyl dithiocarbonates were created, although the reversed addition excludes homolytic substitution at phosphorus (Scheme 10) [36].

**Table 4:** Thiophosphination of terminal alkynes.

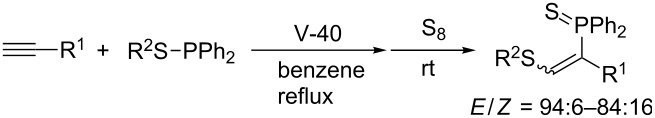		

R^1^	R^2^	Yield (%)

C_10_H_21_	Ph	75
*c*-C_6_H_11_	Ph	61
Ph	Ph	83
C_6_H_4_-*p*-OMe	Ph	75
C_6_H_4_-*o*-OMe	Ph	85
C_6_H_4_-*p*-COMe	Ph	69
C_6_H_4_-*p*-CO_2_Me	Ph	73
C_6_H_4_-*p*-CF_3_	Ph	69
C_6_H_4_-*p*-NH_2_	Ph	80
(CH_2_)_3_OH	Ph	66
C_6_H_4_-*p*-OMe	C_12_H_25_	70
C_10_H_21_	C_12_H_25_	42
C_10_H_21_	*t*-Bu	51

**Scheme 11:**
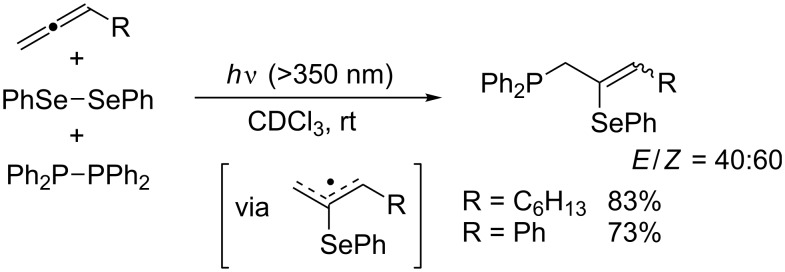
Photoinduced selenophosphination of allenes.

**Scheme 12:**
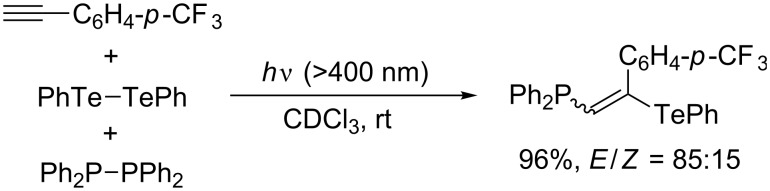
Photoinduced tellurophosphination.

Kawaguchi, Nomoto, and Ogawa seminally studied the photoinduced radical chalcogenophosphination of alkynes and allenes by means of PhCh–ChPh/Ph_2_P–PPh_2_ binary systems (Ch = S, Se, Te) [30,37–39]. The regioselective outcome of the photoinduced thio- and selenophosphination of terminal alkynes (Table 5) is similar to that of the thermal thiophosphination (Scheme 9). Detailed mechanistic studies revealed that comproportionation between PhSe–SePh and Ph_2_P–PPh_2_ occurs smoothly to generate PhSe–PPh_2_ as the actual reactive species. Selenophosphination of terminal allene affords (2-phenylselenyl-2-alkenyl)diphenylphosphine regioselectively (Scheme 11). Notably, the sense of the regioselectivity of tellurophosphination by a PhTe–TePh/Ph_2_P–PPh_2_ system is opposite to those of the thio- and selenophosphination (Scheme 12). This reversal indicates that homolytic substitution at tellurium overwhelms that at phosphorus and that a diphenylphosphanyl radical is more reactive than a phenyltelluranyl radical.

**Table 5:** Photoinduced thio- and selenophosphination by dichalcogen/diphosphine binary system.

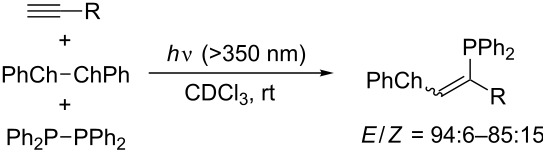		

R	Ch	Yield (%)

C_6_H_13_	S	77
C_6_H_4_-*p*-OMe	S	91
1-cyclohexenyl	S	87
C_6_H_4_-*p*-Br	Se	96
C_6_H_4_-*p*-OMe	Se	78
1-cyclohexenyl	Se	83

### Substitution of halides (X), carboxys (COOR), or carboxylates (OCOR) with phosphorus

**Scheme 13:**
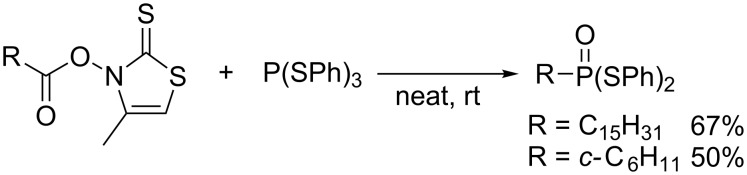
Decarboxylative phosphorylation of carboxylic acid derivatives.

**Scheme 14:**
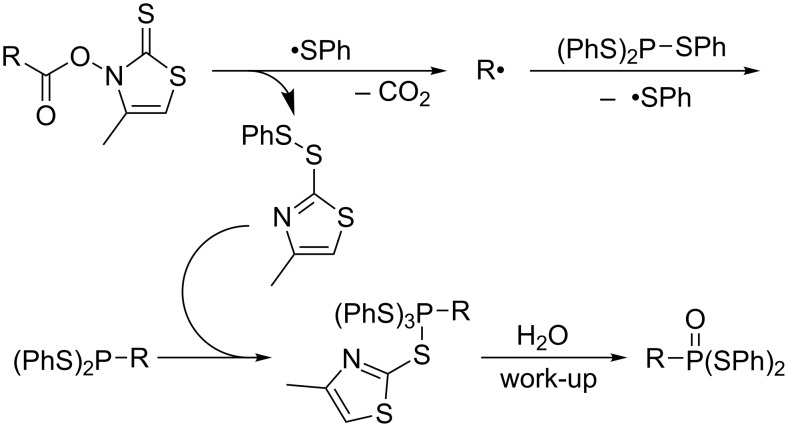
Plausible mechanism of decarboxylative phosphorylation.

After scattered research efforts into the uncontrolled radical C–H phosphination under harsh reaction conditions [40], Barton elegantly devised radical decarboxylative phosphorylation of carboxylic thiohydroxamic mixed anhydrides (Scheme 13) [41]. Radical addition of a phenylsulfanyl radical to the thiocarbonyl generates the corresponding alkyl radical R•, which undergoes homolytic substitution at the phosphorus of P(SPh)_3_ to furnish (PhS)_2_P–R as the initial product (Scheme 14). Oxidative addition of the disulfide byproduct to the initial product furnishes a pentavalent phosphorus species that is eventually hydrolyzed to an *S*,*S*-diphenyl dithiophosphonate upon workup.

**Scheme 15:**
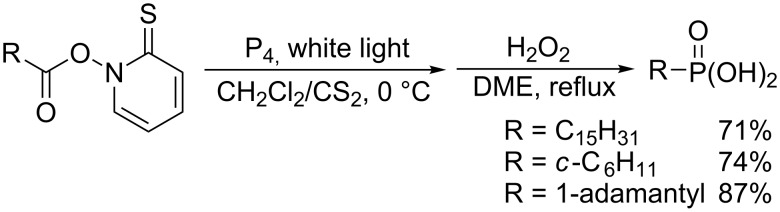
Radical phosphination of PTOC esters with white phosphorus.

Barton also reported that white phosphorus reacts with *N*-acyloxythiopyridones, so-called Barton PTOC esters (Scheme 15) [42]. Photolysis of the esters in the presence of white phosphorus followed by oxidation with hydrogen peroxide yields alkylphosphonic acid. The efficient phosphination would stem from the highly strained structure and the weak P–P bonds of white phosphorus.

**Scheme 16:**
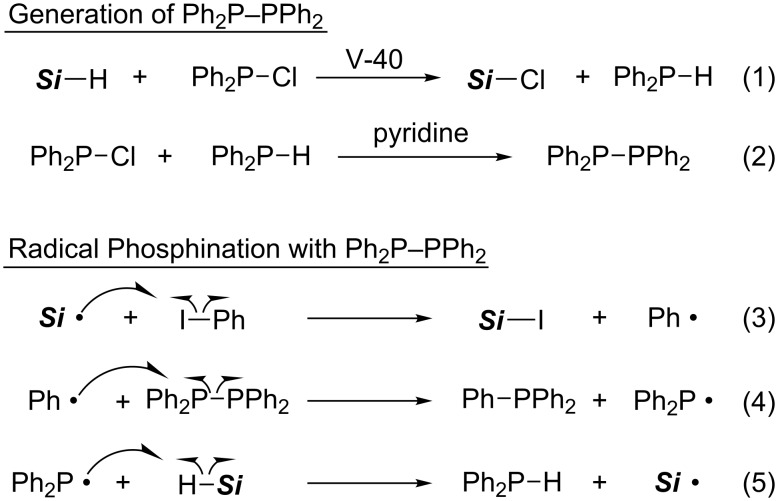
Plausible mechanism of radical phosphination (***Si*** = (Me_3_Si)_3_Si).

After 13 years of silence, radical substitution reactions of organic halides and related compounds with phosphination agents have now been rapidly developing since 2006. Yorimitsu and Oshima invented radical phosphination of organic halides with tetraphenyldiphosphine (Table 6) [43]. Tetraphenyldiphosphine is generated in situ by radical reduction of chlorodiphenylphosphine with tris(trimethylsilyl)silane followed by condensation of the resulting diphenylphosphine with the remaining chlorophosphine (Scheme 16, equation 1 and 2). An aryl radical reacts with tetraphenyldiphosphine to liberate a diphenylphosphanyl radical, which abstracts hydrogen from tris(trimethylsilyl)silane to sustain the chain propagation (Scheme 16, equation 3–5). The in situ formations of diphenylphosphine and of tetraphenyldiphosphine can exclude the handling of pyrophoric diphenylphosphine and air-sensitive tetraphenyldiphosphine. The user-friendly conditions are also suitable for dicyclohexylphosphination with ClP(*c*-C_6_H_11_)_2_.

**Table 6:** Radical phosphination of aryl iodides.

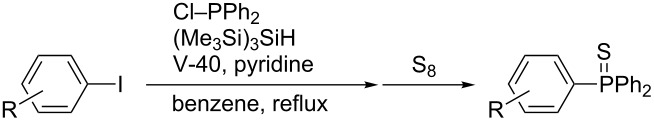	

R	Yield (%)

H	88
2,4,6-Me_3_	63
2-MeO	65
4-MeO	75
4-CF_3_	78
4-Br	66
4-CN	69
4-COCH_3_	47
4-OTf	68
4-CO_2_CH_2_CH=CH_2_	78

**Scheme 17:**
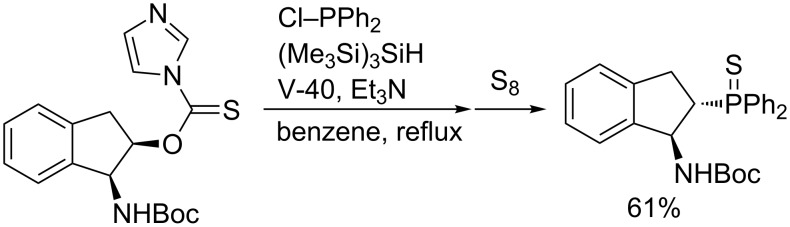
Stereoselective phosphination leading to (*S*,*S*)-aminophosphine derivative.

Phosphination of alkyl halides as substrates results in unsatisfactory yields. Instead, Barton’s alkyl imidazole-1-carbothioates are good substrates for this radical phosphination (Table 7). Conversion of an optically pure *cis*-carbothioate leads to *trans*-aminophosphine of potential use as a ligand (Scheme 17). Diphosphine approaches the resulting radical from the opposite side of the NHBoc group to invert the original stereochemistry.

**Table 7:** Radical phosphination of alkyl imidazole-1-carbothioates.

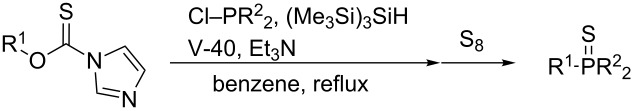		

R^1^	R^2^	Yield (%)

*c*-C_6_H_11_	Ph	87
*c*-C_6_H_11_	*c*-C_6_H_11_	68
EtOCOCH_2_CH(CH_3_)	Ph	89
3-oxacyclopentyl	Ph	87
C_6_H_13_	Ph	63

**Figure 1:**
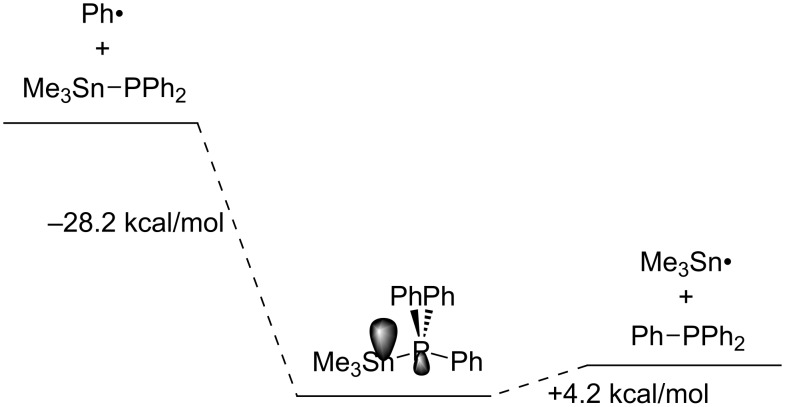
Calculated reaction profile of homolytic substitution between Ph• and Me_3_Sn–PPh_2_ at the B2-PLYP-D/TZVVP//PBE-D/TZVP level. Gray lobes indicate major spin densities.

Studer developed in 2007 new elegant reagents Me_3_Sn–PPh_2_ and Me_3_Si–PPh_2_ for radical phosphination [44]. The scope of his phosphination with Me_3_Sn–PPh_2_ is wide as summarized in Table 8. Although the low toxicity of Me_3_Si–PPh_2_ is advantageous, phosphination with Me_3_Si–PPh_2_ is limited to alkyl halides or imidazole-1-carbothioate. Density functional theory calculations have clarified the homolytic substitution process is a two-step mechanism via a tetracoordinated phosphorus atom (Figure 1). The spin density in the tetracoordinated phosphorus intermediate is localized mostly on the Sn atom while the remaining spin density is found in the equatorial position of the distorted trigonal prismatic P atom.

**Table 8:** Radical phosphination with Me_3_Sn–PPh_2_.

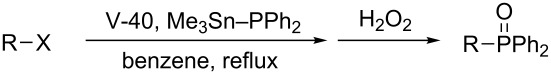	

R–X	Yield (%)

*p*-MeOC_6_H_4_–I	73
*p*-NCC_6_H_4_–I	79
*p*-CF_3_C_6_H_4_–I	75
*p*-ClC_6_H_4_–I	72
*o*-MeOC_6_H_4_–I	59
*o*-MeO_2_CC_6_H_4_–I	73
CH_3_CBr=CH_2_	76
C_5_H_11_–I	79
*c*-C_6_H_11_–I	94
C_11_H_23_–Br	54
*t*-Bu–Br	83
C_5_H_11_–SePh	60
*c*-C_6_H_11_–OCS-1-imidazole	57

**Scheme 18:**
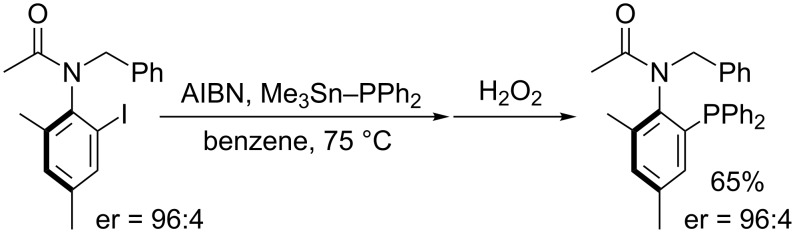
Phosphination with retention of axial chirality.

**Scheme 19:**
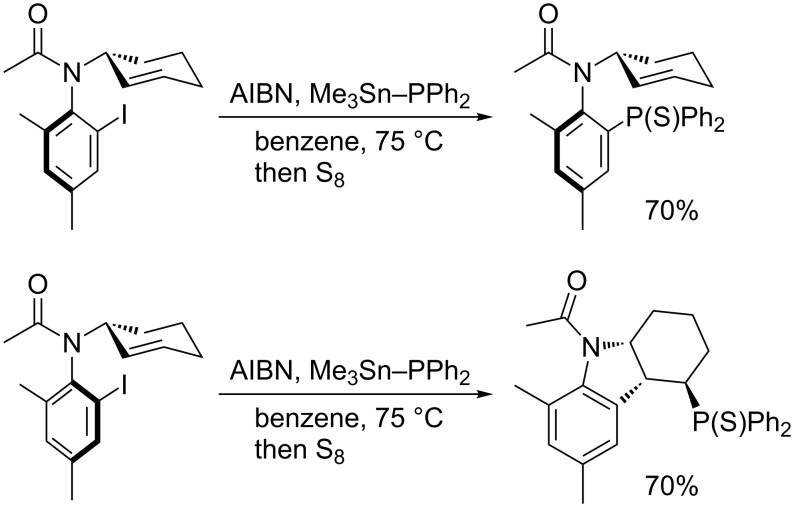
Chemodivergent phosphination.

The rate constant for phosphination of an aryl radical with Me_3_Sn–PPh_2_ is calculated to be ca. 9 × 10^8^ M^−1^s^−1^ by competition kinetics with Bu_3_SnH reduction [45]. This large rate constant allows for stereospecific trapping of axially chiral acyl radicals with Me_3_Sn–PPh_2_ (Scheme 18). Chemodivergent trapping of diastereomers of an *N*-(2-cyclohexenyl)acetanilide derivative is interesting (Scheme 19). One isomer undergoes direct phosphination while the other cyclizes prior to phosphination.

Intermolecular phosphinative radical addition of alkyl iodides to activated alkenes proceeds in the presence of Me_3_M–PPh_2_ (M = Sn, Si) and V-40 (Table 9) [26]. Secondary and tertiary alkyl iodides participate in the addition reaction while primary alkyl iodide results in direct phosphination prior to the expected addition. Not only acrylate ester but also acrylamide, vinyl sulfone, and acrylonitrile are good radical acceptors in this addition.

**Table 9:** Phosphinative radical addition of alkyl iodides to activated alkenes.

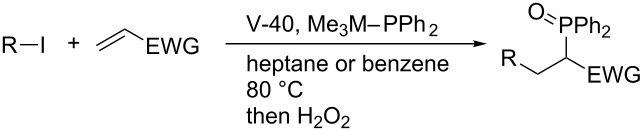			

R	*M*	EWG	Yield (%)

*c*-C_6_H_11_	Si	CO_2_*t*-Bu	72
*c*-C_6_H_11_	Sn	CO_2_*t*-Bu	64
*t*-Bu	Si	CO_2_*t*-Bu	76
*t*-Bu	Sn	CO_2_*t*-Bu	69
C_5_H_11_	Si	CO_2_*t*-Bu	<5
C_5_H_11_	Sn	CO_2_*t*-Bu	<5
*c*-C_6_H_11_	Sn	SO_2_Ph	48
*c*-C_6_H_11_	Sn	CONMe_2_	44
*c*-C_6_H_11_	Sn	CN	79

**Scheme 20:**
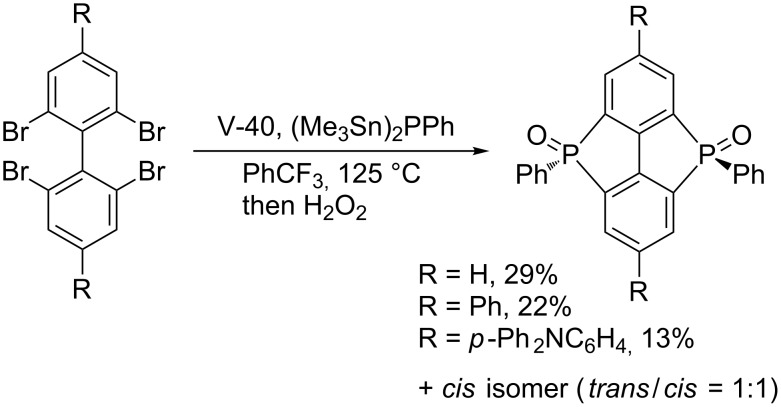
Bis(phosphoryl)-bridged biphenyls by radical phosphination.

**Scheme 21:**
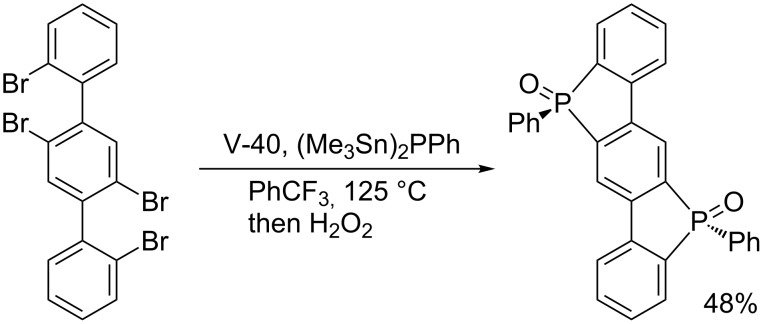
Bis(phosphoryl)-bridged ladder triphenylene by radical phosphination.

Studer’s stannylphosphine technology is reliable enough to be applied to the construction of interesting π-conjugated frameworks. In collaboration with Yamaguchi, Studer invented a new radical reagent (Me_3_Sn)_2_PPh for the synthesis of highly strained bis(phosphoryl)-bridged biphenyls (Scheme 20) [46]. Subsequently, Liu reported an efficient synthesis of bis(phosphoryl)-bridged ladder triphenylene by means of the radical clipping with (Me_3_Sn)_2_PPh (Scheme 21) [47]. In light of the increasing importance of phosphoryl-bridged π-conjugated skeletons in organic material sciences, (Me_3_Sn)_2_PPh will serve as a key reagent.

**Scheme 22:**
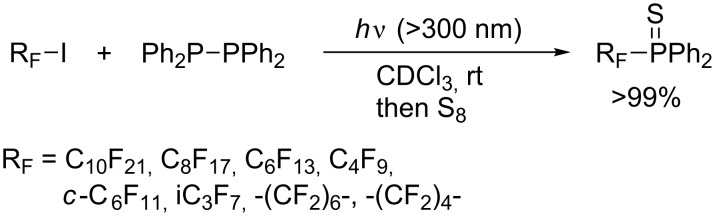
Photoinduced phosphination of perfluoroalkyl iodides with tetraphenyldiphosphine.

Ogawa developed photoinduced phosphination of perfluoroalkyl iodides with tetraorganodiphosphines (Scheme 22) [48]. Remarkably, the phosphination proceeds quantitatively. The phosphine ligands thus synthesized are fluorophilic. Particularly, two molecules of perfluorodecyldiphenylphosphine coordinate to palladium dichloride to form a catalytically active palladium complex that is useful for a fluorous/organic biphasic system.

**Scheme 23:**
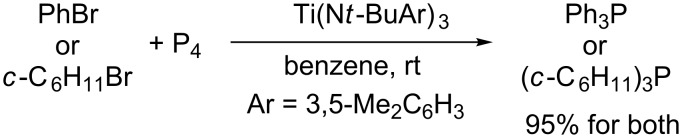
Ti(III)-mediated radical phosphination of organic bromides with white phosphorus.

Cummins devised radical phosphination of bromobenzene or bromocyclohexane with white phosphorus by means of a trivalent titanium complex (Scheme 23) [49]. This represents a unique direct method for preparing triorganophosphine without recourse to any trivalent phosphorus sources such as PCl_3_.

## Conclusion

Introduction of a phosphorus atom by a radical process has offered an intriguing tool for the synthesis of organophosphorus compounds. Radical addition of a phosphorus-centered radical has been representative so far. A recent dramatic growth in reports of homolytic substitution at phosphorus in organic synthesis has changed the landscape of radical phosphination. Radical addition that involves homolytic substitution at phosphorus always culminates in difunctionalization of a multiple bond. Therefore this methodology will find application in the synthesis of complex phosphines including bidentate ones. Radical substitution of halogen in organic halide with phosphorus will be an alternative to classical ionic substitution. Advantageously, it requires neither highly basic conditions nor transition metals. Homolytic substitution at phosphorus is still in its infancy. In light of the rich chemistry of organophosphorus compounds, it will find wider application in organic synthesis in the future.
